# Assessment of No-Reflow in Patients With STEMI After Intracoronary Tirofiban After Opening of the Vessel

**DOI:** 10.14740/cr2180

**Published:** 2026-02-28

**Authors:** Mohammed Ali Mohammed Hammad, Wael Anwar Elshahat Hassib, Mohamed Kamal Ibrahim Salama, Husna Irfan Thalib, Mohammed Moanes, Muhammad Reihan

**Affiliations:** a Cardiology Department, Faculty of Medicine, Kafr Elsheikh University, Kafr Elsheikh, Egypt; b General Medicine Practice Program, Batterjee Medical College, Jeddah, Saudi Arabia; c Cardiology Department, Faculty of Medicine, Al-Azhar University, Cairo, Egypt; d Hayat National Hospitals, Saudi Arabia; e Cardiology Department, Faculty of Medicine, Al-Azhar University, Damietta, Egypt; f Department of Internal Medicine, Batterjee Medical College, Jeddah 21442, Saudi Arabia

**Keywords:** Glycoprotein IIb/IIIa inhibitor, Intracoronary, No-reflow phenomenon, Percutaneous coronary intervention, ST-segment elevation myocardial infarction, Tirofiban

## Abstract

**Background:**

No-reflow phenomenon (NRP) following primary percutaneous coronary intervention (PPCI) remains a critical determinant of adverse outcomes in ST-segment elevation myocardial infarction (STEMI) cases despite successful epicardial recanalization. The core purpose of this study was to establish the value of intracoronary (IC) tirofiban, delivered via the IC route, in mitigating the occurrence of NRP for STEMI cases subsequent to successful vessel reopening.

**Methods:**

This randomized controlled double-blind study enrolled 60 STEMI cases. Following successful PCI, cases with thrombolysis in myocardial infarction (TIMI) flow grade less than 3 were randomized to receive either IC tirofiban (25 ug/kg) or saline 0.9% as placebo, in addition to standard pre-procedural therapy with aspirin, heparin, and ticagrelor. TIMI flow grade and incidence of NRP were evaluated. Additionally, ST-T normalization in electrocardiogram (ECG) was assessed. Bleeding complications and major adverse cardiac events (MACEs) were recorded during hospitalization and at 30-day follow-up.

**Results:**

The tirofiban group demonstrated notably superior coronary flow restoration with 80% achieving TIMI 3 flow versus 46.67% in controls (P = 0.007). NRP occurred in 20% of tirofiban cases compared to 53.33% in controls (P = 0.007). Minor bleeding complications increased in the tirofiban group (26.67% versus 3.33%, P = 0.026), while major bleeding remained absent in both groups. Total in-hospital MACEs were notably reduced with tirofiban treatment compared to controls (3.33% versus 30%, P = 0.012).

**Conclusions:**

In STEMI cases following PPCI, IC tirofiban administration effectively reduces NRP, improves coronary flow restoration, and reduces MACE despite increased minor bleeding risk.

## Introduction

Acute ST-segment elevation myocardial infarction (STEMI), a critical cardiovascular emergency caused by thrombotic occlusion following plaque rupture or erosion, is best treated with primary percutaneous coronary intervention (PPCI), which offers superior outcomes over thrombolytic therapy [1]. Nevertheless, a sequence of harmful processes, known together as ischemia-reperfusion injury, is paradoxically set in motion by the re-establishment of coronary blood flow to formerly ischemic heart tissue [2].

The pathophysiological mechanisms underlying myocardial ischemia-reperfusion injury encompass a complex interplay of cellular death pathways, including apoptosis, necrosis, and autophagy, alongside maladaptive processes such as cardiomyocyte hypertrophy, interstitial fibrosis, and aberrant angiogenesis [3]. These molecular events manifest clinically as diverse phenomena such as lethal reperfusion trauma, microvascular impairment, myocardial dysfunction, and arrhythmias induced by reperfusion, all of which can substantially compromise patient outcomes despite successful epicardial recanalization [4].

Microvascular obstruction, a common and serious complication after coronary reperfusion for acute myocardial infarction, involves structural and functional damage to the coronary microvasculature, leading to a no-reflow phenomenon (NRP) despite successful epicardial reperfusion [5]. The NRP has been identified as one of the primary determinants of adverse cardiovascular outcomes in cases with STEMI, notably impacting short and long-term prognosis [6].

Successful recanalization of the infarct-related artery (IRA) with restoration of normal coronary flow is fundamentally associated with preserved left ventricular function and reduced mortality rates in cases with STEMI [7]. Conversely, the occurrence of NRP notably attenuates the clinical benefits derived from successful epicardial recanalization, highlighting the critical importance of maintaining optimal microvascular perfusion [8].

Contemporary therapeutic approaches have increasingly focused on both mechanical and pharmacological interventions to optimize myocardial reperfusion, with particular emphasis on the adjunctive use of glycoprotein IIb/IIIa inhibitors in cases undergoing PPCI [9]. While current clinical practice guidelines recommend the administration of small molecule glycoprotein IIb/IIIa inhibitors as upstream therapy followed by continuous infusion in selected STEMI cases, evolving clinical practices and newer antiplatelet strategies have influenced the utilization patterns of these agents [10]. Accumulated evidence from multiple clinical investigations has demonstrated that both intravenous (IV) and intracoronary (IC) administration of glycoprotein IIb/IIIa inhibitors can notably improve clinical outcomes and reduce mortality in cases with STEMI [11, 12].

Tirofiban represents a small molecule nonpeptide tyrosine derivative classified within the glycoprotein IIb/IIIa inhibitor family [13]. Regarded as the most potent available inhibitors of platelet aggregation, these agents operate by competitively preventing both fibrinogen and von Willebrand factor from attaching to the glycoprotein IIb/IIIa receptor complex located on the platelet surface [14].

The IC administration of tirofiban offers theoretical and practical advantages over systemic delivery by achieving higher local concentrations at the thrombus site, enhancing receptor occupancy and platelet inhibition, and thereby promoting more effective thrombus resolution [15].

Given these considerations, the present investigation seeks to evaluate the incidence and determinants of NRP in cases with STEMI following IC administration of glycoprotein IIb/IIIa inhibitors after successful epicardial vessel recanalization.

## Materials and Methods

This randomized controlled double-blinded study was conducted on 60 individuals aged 18 years or older of both sexes presenting with STEMI at Kafr Elsheikh University Hospital, Egypt from May 2025 to October 2025.

A diagnosis of STEMI was established when cases presented with typical chest pain lasting in excess of 30 min, accompanied by either an ST-segment elevation surpassing 1 mm in at least two adjacent electrocardiographic leads or the acute onset of a left bundle branch block [16].

The study was approved by the institutional ethical committee (ID: KFSIRB200-587), registered on ClinicalTrials.gov (ID: NCT06966674). Informed written consent was obtained from all participants.

Exclusion criteria were prior thrombolytic therapy within 24 h, known malignancies, thrombocytopenia, end-stage liver disease, cardiogenic shock, renal failure with a glomerular filtration rate below 30 mL/min, or any contraindication to tirofiban administration.

### Randomization and blindness

Random allocation was performed using an online randomization program [17] to generate the randomization sequence. Randomization was stratified considering age, sex, IRA, and comorbidities to ensure balanced clinical characteristics between groups. Individual patient codes were secured in opaque sealed envelopes to maintain allocation concealment. Participants were randomly assigned in a 1:1 ratio using parallel group allocation to one of two treatment arms. The study group (tirofiban group, n = 30) received IC tirofiban (Aggrastat^®^, Correvio LLC, USA) at a dose of 25 µg/kg, while the control group (placebo group, n = 30) received IC 0.9% saline solution as placebo. The selected dose of 25 µg/kg for IC tirofiban was based on prior studies demonstrating effective platelet inhibition with minimal systemic bleeding risk. A rigorous double-blinding methodology was implemented wherein both the cases and the outcome assessors remained unaware of group allocation throughout the study period. The interventional medications were prepared by a pharmacist who was not involved in subsequent patient management or data collection. The syringes were labeled only with the patient’s study number and were identical in appearance, volume, and color, thereby maintaining the integrity of the blinding process. Tirofiban was administered as a single IC bolus without subsequent prolonged IV infusion.

Comprehensive medical and surgical histories were obtained from all participants. Physical examinations were performed and routine laboratory investigations were conducted according to standard protocols prior to the intervention. An electrocardiogram (ECG) was done to assess the ST segment shift either elevation or depression, rhythm assessment, and T wave changes.

### PPCI protocol

All cases received standardized pre-procedural medication involved a 300 mg oral dose of aspirin, IV heparin at 70 U/kg, and a 180 mg oral ticagrelor loading dose. Within the cardiology catheterization laboratory, all PPCI procedures were performed utilizing the femoral approach as the sole access site. In situations permitting, direct stenting constituted the chosen intervention technically feasible, with balloon pre-dilatation reserved for cases where direct stenting was not appropriate. The intervention was limited to the IRA only.

A residual stenosis of less than 50% and the presence of thrombolysis in myocardial infarction (TIMI) grade 3 flow post-intervention indicated procedural success. Conversely, NRP was characterized by post-procedural TIMI flow grades of 0, 1, or 2. All procedures used femoral access due to operator experience and catheterization lab constraints. No radial procedures were performed. All bleeding complications were categorized by access site; no non-access site major bleeding occurred.

### Management of NRP and study intervention

Following stent implantation, cases demonstrating TIMI flow grade less than 3 underwent additional post-dilatation procedures with balloon catheters. IC vasodilator agents, including nitrates and diltiazem, were administered in clinically appropriate cases. Cases who continued to exhibit TIMI flow grade less than 3 despite these interventions were classified as having NRP and subsequently randomized to receive either the active treatment or placebo.

The study intervention involved administration of either IC tirofiban (25 µg/kg) or saline solution 0.9% as placebo through the guiding catheter into the IRA. For lesions located in the circumflex or left anterior descending arteries, the intervention was delivered from the left main coronary artery. To assess the acute effects of tirofiban on coronary NRP, the bolus dose was administered directly into the IC circulation following successful recanalization of the thrombotic occlusion.

Repeat coronary angiography was performed 10 min following the intervention, and TIMI flow grades were recorded for both treatment groups.

### Outcome measures and assessment criteria

Coronary blood flow was assessed using TIMI flow grades and TIMI frame count before and within 15 min after treatment [18]. The TIMI bleeding criteria served as the basis for categorizing hemorrhagic complications, where major bleeding was identified by the occurrence of intracranial hemorrhage or a hemoglobin reduction surpassing 50 g/L, while minor bleeding encompassed visible bleeding events with a hemoglobin drop > 30 g/L. A platelet count less than 60 × 10^9^/L was utilized to define thrombocytopenia.

The occurrence of major adverse cardiac events (MACEs) in addition to post-infarction angina and repeat revascularization was noted throughout hospitalization and again at the 30-day follow-up.

The primary outcome was coronary artery blood flow evaluated by TIMI flow grade and frame count pre- and post-treatment. TIMI flow grading was performed independently by two blinded interventional cardiologists, and disagreements were resolved by consensus. Secondary outcome was incidence of MACE within 30 days post-intervention. Left ventricular function was assessed by transthoracic echocardiography at baseline and at 30-day follow-up to monitor changes in ejection fraction and wall motion abnormalities.

### Sample size calculation

G*Power 3.1.9.2 (Universitat Kiel, Germany) was employed for sample size estimation. Previous research [19] indicated mean ± SD IRA blood flow, as measured by the enhanced TIMI frame count method, to be 1.68 ± 0.23 mL/s in the tirofiban group and 1.42 ± 0.31 mL/s in the control group. The sample size was derived using an effect size of 0.953, a 95% confidence limit, 90% power for the study, and a 1:1 group ratio. To accommodate for potential participant attrition, five additional cases were incorporated into each group, leading to a planned recruitment of 30 cases per group.

### Statistical analysis

SPSS v29 (IBM^©^, Armonk, NY, USA) was the software employed for all statistical procedures. The normality of data distribution was evaluated using the Shapiro-Wilk test in conjunction with histograms. Quantitative parametric data were presented as mean and SD and subsequently analyzed utilizing the unpaired Student’s *t*-test. Qualitative variables were detailed by frequency and percentage, with their analysis performed using the Chi-square test or Fisher’s exact test where applicable. A two-tailed P value ≤ 0.05 was pre-determined as the criterion for statistical significance.

## Results

**Figure 1:**
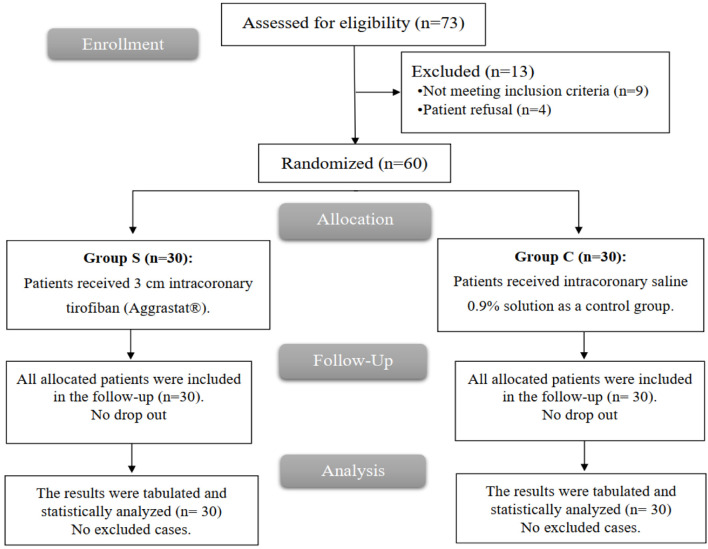
CONSORT flowchart of the enrolled patients.

A total of 73 cases were screened for potential enrollment in the study. Of these, nine did not fulfill the specified criteria, and four elected not to participate. Consequently, the remaining 60 cases were randomly assigned to one of two groups, with each placebo group containing 30 cases. Comprehensive follow-up and statistical analysis were conducted for all allocated cases (Fig. 1).

Age, sex, BMI, comorbidities, or laboratory parameters including hemoglobin, creatinine, liver enzymes, lipid profile, and troponin-I were similar across groups (Table 1).

**Table 1:** Pre-Interventional Data and Laboratory Tests

	Tirofiban group (n = 30)	Placebo group (n = 30)	P value
Age (years)	59.17 ± 14.65	57.27 ± 13.21	0.600
Sex			0.559
Male	21 (70%)	23 (76.67%)	
Female	9 (30%)	7 (23.33%)	
Weight (kg)	83.37 ± 9.93	80.8 ± 8.55	0.288
Height (cm)	171.77 ± 5.7	170.3 ± 5.85	0.330
BMI (kg/m^2^)	28.34 ± 3.86	27.97 ± 3.73	0.712
Comorbidities			
Hypertension	14 (46.67%)	11 (36.67%)	0.432
Diabetes mellitus	10 (33.33%)	8 (26.67%)	0.573
Smoking	3 (10%)	5 (16.67%)	0.707
Hemoglobin (g/dL)	12.82 ± 1.82	13.16 ± 2.06	0.509
Platelet (10^3^/µL)	247.97 ± 66.7	253.03 ± 66.42	0.769
Creatinine (mg/dL)	1.01 ± 0.5	1.18 ± 0.36	0.137
Urea (mg/dL)	20.93 ± 8.64	22.07 ± 9.44	0.629
ALT (U/L)	32.2 ± 12.19	34.7 ± 15.14	0.484
AST (U/L)	41.63 ± 18.73	47.33 ± 22.53	0.291
Total cholesterol (mg/dL)	184.4 ± 43.08	189.23 ± 39.32	0.652
Triglycerides (mg/dL)	112.07 ± 46.96	113.17 ± 53.13	0.933
LDL (mg/dL)	120.57 ± 31.09	125.87 ± 40.34	0.571
HDL (mg/dL)	38.8 ± 12.71	34.9 ± 13.88	0.261
Troponin-I (ng/mL)	8.24 ± 4.21	9.53 ± 5.1	0.292

Data presented as mean ± SD or frequency (%). ALT: alanine transaminase; AST: aspartate aminotransferase; BMI: body mass index; HDL: high-density lipoprotein; LDL: low-density lipoprotein.

No substantial differences were observed between the two groups in the IRA, balloon predilation, stent length, stent diameter, and stent inflation pressure. All cases in both groups needed stent implantation (Table 2).

**Table 2:** Interventional Data

	Tirofiban group (n = 30)	Placebo group (n = 30)	P value
Infarct-related artery			0.856
Left anterior descending	14 (46.67%)	13 (43.33%)	
RCA	7 (23.33%)	6 (20%)	
Circumflex	9 (30%)	11 (36.67%)	
Stent implantation	30 (100%)	30 (100%)	-
Balloon predilatation	20 (66.67%)	25 (83.33%)	0.136
Stent length (mm)	24.8 ± 4.87	24.2 ± 4.8	0.633
Stent diameter (mm)	3.22 ± 0.48	3.12 ± 0.38	0.409
Stent inflation pressure (atm)	14.37 ± 2.5	14.8 ± 3.68	0.596

Data presented as mean ± SD or frequency (%). RCA: right coronary artery.

**Figure 2:**
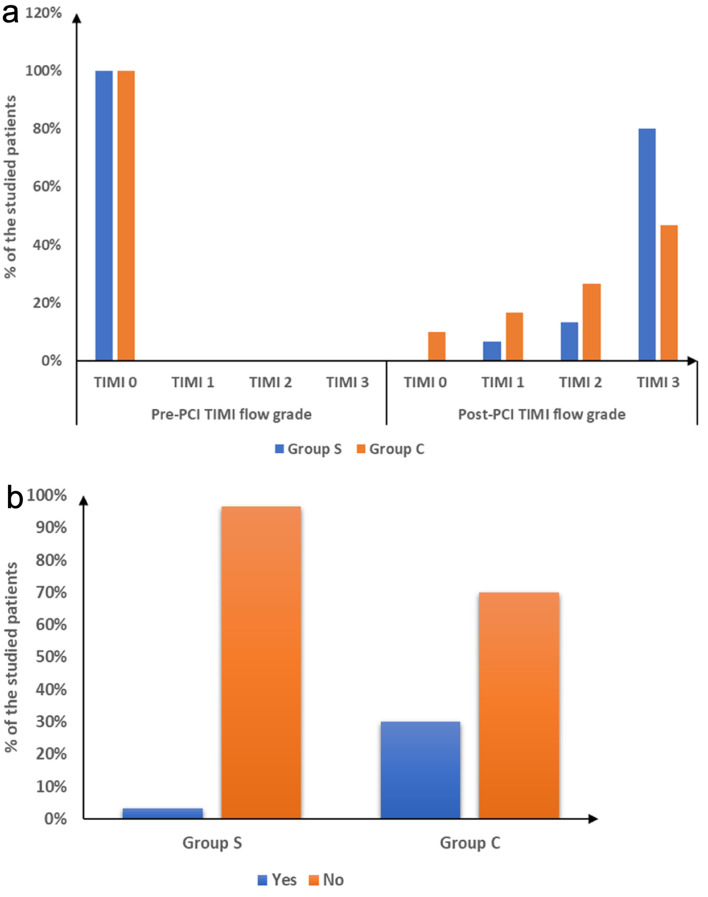
(a) Pre- and post-PCI TIMI flow grade and (b) total in hospital TVF of the studied groups. PCI: percutaneous coronary intervention; TIMI: thrombolysis in myocardial infarction; TVF: target vessel failure.

**Figure 3:**
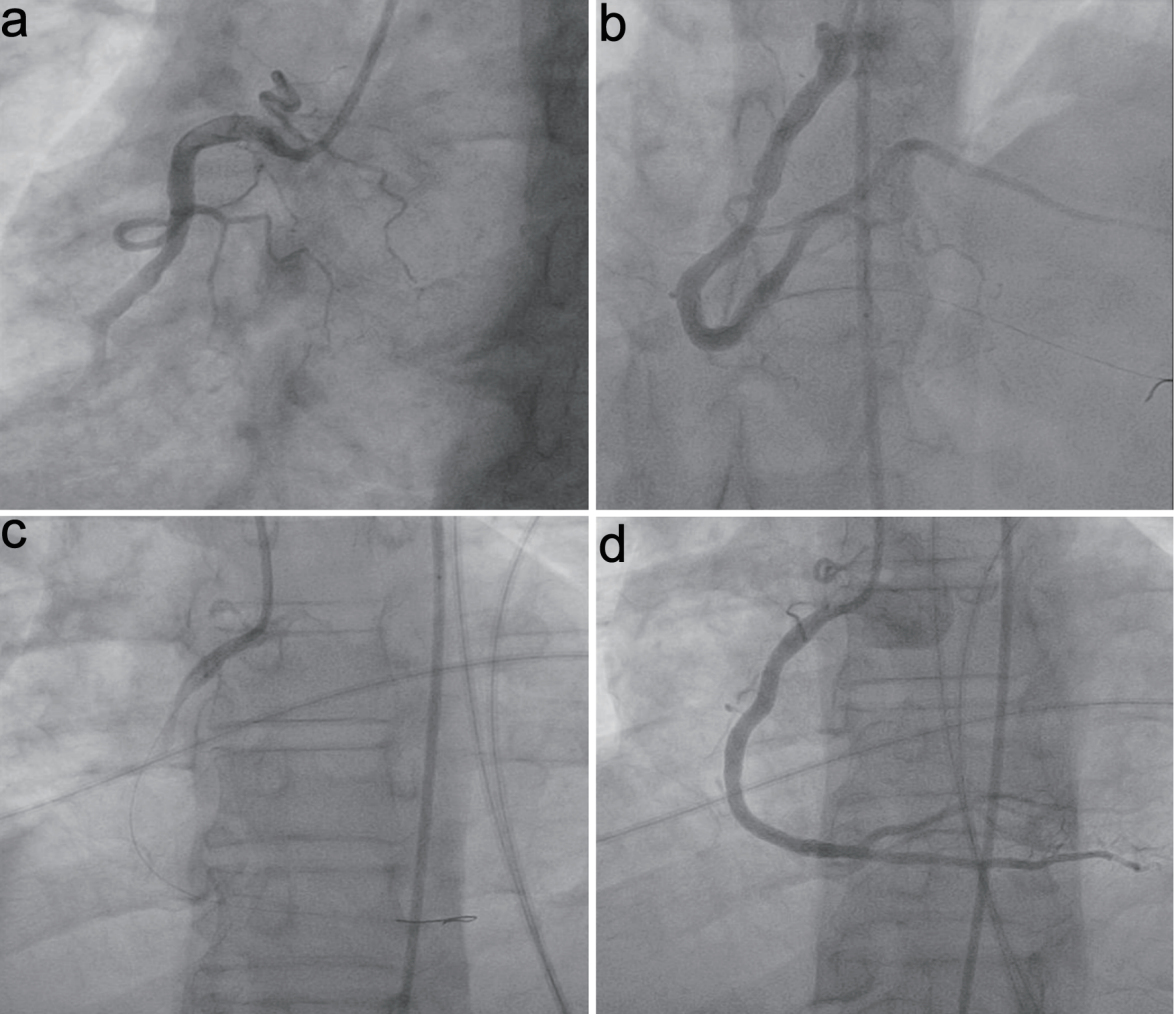
Representative angiographic images of the right coronary artery (RCA) in STEMI patients. (a) Pre-PCI, placebo group. (b) Post-PCI, placebo group. (c) Pre-PCI, IC tirofiban group. (d) Post-PCI, IC tirofiban group, demonstrating improved TIMI 3 flow with tirofiban administration. IC: intracoronary; PCI: percutaneous coronary intervention; TIMI: thrombolysis in myocardial infarction.

Pre-PCI TIMI flow was grade 0 in all cases. Post-tirofiban, TIMI flow grade, reflow, and ST-T normalization were notably higher in group S than group C (P = 0.041, 0.007, and 0.034). Treatment for no-reflow post-PCI (IC nitrate and diltiazem) was notably lower in group S (P = 0.007). Post-PCI balloon dilatation showed no substantial difference between groups. Minor bleeding was notably higher in group S (P = 0.026), while major bleeding did not occur in either group. Cardiac death was not notably different between groups. Total in-hospital target vessel failure (TVF) was notably lower in group S compared to group C (P = 0.012) (Table 3, Fig. 2). Representative angiographic images of the right coronary artery (RCA) before and after PCI, with and without IC tirofiban, are shown in Figure 3. Post-tirofiban administration, TIMI 3 flow was achieved, illustrating improved coronary perfusion compared to the placebo group.

**Table 3:** TIMI Flow Grade, ST-T Normalization, Treatment Outcomes, and Complications

	Group S (n = 30)	Group C (n = 30)	P value
Pre-PCI TIMI flow grade			-
TIMI 0	30 (100%)	30 (100%)	
Post-PCI TIMI flow grade			0.041
TIMI 0	0 (0%)	3 (10%)	
TIMI 1	2 (6.67%)	5 (16.67%)	
TIMI 2	4 (13.33%)	8 (26.67%)	
TIMI 3	24 (80%)	14 (46.67%)	
Post tirofiban reflow			0.007
Reflow	24 (80%)	14 (46.67%)	
No reflow	6 (20%)	16 (53.33%)	
ST-T normalization			0.034
Complete resolution	26 (86.67%)	17 (56.67%)	
Partial resolution	2 (6.67%)	5 (16.67%)	
Poor resolution	2 (6.67%)	8 (26.67%)	
Treatment for no-reflow post PCI			
Intracoronary nitrate	6 (20%)	14 (46.67%)	0.007
Intracoronary diltiazem	6 (20%)	14 (46.67%)	0.007
Post-PCI balloon dilatations	15 (50%)	16 (53.33%)	0.796
Complications			
Minor bleeding	8 (26.67%)	1 (3.33%)	0.026
Major bleeding	0 (0%)	0 (0%)	-
Cardiac death	0 (0%)	2 (6.67%)	0.492
Total in hospital TVF	1 (3.33%)	9 (30%)	0.012

Data presented as frequency (%). PCI: percutaneous coronary intervention; TIMI: thrombolysis in myocardial infarction; TVF: target vessel failure.

## Discussion

The management of STEMI through PPCI represents a critical therapeutic intervention, yet the occurrence of NRP continues to pose substantial challenges that adversely impact patient outcomes and long-term prognosis [20, 21].

This study shows that IC tirofiban notably improved coronary flow with higher TIMI 3 flow rates in STEMI cases, matching the meta-analysis by Shi et al [22], which demonstrated that IC low-dose tirofiban notably improved TIMI flow grade compared to IV administration (P = 0.037). Similarly, Akpek et al [16] reported successful reperfusion in 32% of cases receiving IC tirofiban compared to only 10% in the placebo group, with substantial improvement in TIMI flow grade (P < 0.001).

The mechanism underlying these improvements appears related to the direct local delivery of tirofiban to the site of coronary occlusion, allowing for higher local drug concentrations while minimizing systemic exposure [14]. Wang et al [19] corroborated these findings in cases with serious thrombus burden, reporting increased TIMI-3 flow in the IRA (89.3% versus 85.4%, P < 0.05) and enhanced blood flow calculated using TIMI frame count methodology. The superior efficacy of IC versus IV administration was further substantiated by Tang et al [23], who observed improved TIMI flow grading in the IC group (P = 0.022).

The substantial reduction in NRP incidence observed in our study represents a clinically substantial finding that corresponds with existing literature on IC tirofiban efficacy. This improvement directly translates to reduced need for rescue interventions, as evidenced by the decreased requirement for IC nitrate and diltiazem administration (20% versus 46.67% for both interventions). Duan et al [24] found that IC tirofiban notably improved coronary blood flow and myocardial perfusion after NRP, achieving TIMI grade 3 flow in 76.5% of cases versus 52.9% with nitroprusside (P = 0.03).

The temporal aspect of tirofiban administration appears crucial for optimal outcomes, as demonstrated by Mei and Yu [25] who found that timing of tirofiban administration is critical, with delivery during TIMI grade ≥ 1 yielding better blood flow and reduced myocardial injury compared to administration without forward flow. Gao et al [26] further supported these observations, reporting lower incidence of NRP (P = 0.031) when thrombectomy was combined with IC tirofiban administration compared to thrombectomy alone or neither treatment.

Although a lower in-hospital MACE rate was observed in the tirofiban group (3.33% vs. 30%), the study was not powered to detect differences in MACE; these findings should be interpreted as hypothesis-generating. Supporting evidence from Ma et al [27] using cardiac MRI showed that IC tirofiban reduced microvascular obstruction (56% versus 36%, P = 0.004), improved left ventricular strain (−12.5 versus −12.3, P = 0.042), and enhanced myocardial perfusion index (0.11 versus 0.09, P = 0.026), demonstrating superior cardiac outcomes compared to IV administration in cases with myocardial infarction.

The preservation of left ventricular function represents a critical long-term benefit, as demonstrated by Chen et al [28], who reported notably higher left ventricular ejection fraction and lower incidence of MACE in elderly STEMI cases receiving combined IC tirofiban and nicorandil [29]. Tian et al [12] conducted a comprehensive meta-analysis revealing that IC tirofiban notably reduced 30-day MACE (P = 0.028) and improved both in-hospital and 6-month left ventricular ejection fraction compared to IV administration.

The observed increase in minor bleeding complications (26.67% versus 3.33%) in our study population warrants careful consideration within the context of overall risk-benefit assessment. Despite this increase in minor bleeding events, the absence of major bleeding complications in both groups suggests an acceptable safety profile for IC tirofiban administration. This safety pattern aligns with findings from Shi et al [22] which revealed no substantial variation in the incidence of bleeding events between IC and IV tirofiban administration in their meta-analysis.

The bleeding risk associated with IC tirofiban administration must be contextualized against the substantial reduction in MACE and improved coronary perfusion outcomes. Qin et al [20] acknowledged a trend toward increased bleeding risk in their meta-analysis but noted that the data did not reach statistical significance. Similarly, Tian et al [12] reported no substantial difference in in-hospital bleeding or thrombocytopenia between IC and IV tirofiban, supporting its acceptable safety profile alongside clinical benefits.

While IC tirofiban has been previously studied, our investigation confirms its efficacy in a contemporary STEMI cohort and serves as an angiographic proof-of-concept; we acknowledge that novelty is limited. Our study has several limitations. TIMI flow grades and TIMI frame count, although commonly used, have low specificity for detecting microvascular obstruction. This is a single-center study with a small sample size and short follow-up, which may limit generalizability. The 30-day follow-up precludes assessment of long-term outcomes. Reliance on TIMI flow grades may overlook nuanced microvascular dysfunction. Lastly, we did not assess left ventricular function with cardiac MRI or directly measure infarct size, which could provide more precise information on myocardial salvage. Despite observed benefits, IC tirofiban is not guideline-endorsed due to limited large-scale randomized trials, heterogeneity of study designs, and short follow-up in existing literature.

### Conclusions

IC tirofiban (25 µg/kg) notably reduced NRP incidence and improved TIMI 3 flow restoration versus placebo in STEMI cases post-PCI. It lowered in-hospital MACE despite increased minor bleeding. Tirofiban improved TIMI flow, which may indirectly reflect enhanced microvascular perfusion and contributes to improved short-term outcomes after epicardial recanalization, although direct measurement of microvascular function was not performed.

## Data Availability

The data supporting the findings of this study are available from the corresponding author upon reasonable request.
